# *Diospyros kaki* calyx inhibits immediate-type hypersensitivity via the reduction of mast cell activation

**DOI:** 10.1080/13880209.2017.1354386

**Published:** 2017-07-20

**Authors:** Min-Jong Kim, Hae Ran Park, Tae-Yong Shin, Sang-Hyun Kim

**Affiliations:** a CMRI, Department of Pharmacology, School of Medicine, Kyungpook National University, Daegu, Republic of Korea;; b College of Pharmacy, Woosuk University, Jeonju, Republic of Korea

**Keywords:** Active systemic anaphylaxis, immunoglobulin E, passive cutaneous anaphylaxis, histamine, nuclear factor-κB

## Abstract

**Context:***Diospyros kaki* L. (Ebenaceae) fruit is widely distributed in Asia and is known to exert anti-inflammatory and antithrombotic effects.

**Objective:** We evaluated the inhibitory effect of aqueous extract of *D. kaki* calyx (AEDKC) on mast cell-mediated immediate-type hypersensitivity and underlying mechanism of action.

**Materials and methods:** For *in vivo*, ovalbumin (OVA)-induced active systemic anaphylaxis (ASA) and immunoglobulin (Ig) E-mediated passive cutaneous anaphylaxis (PCA) models were used. In the ASA, AEDKC (1–100 mg/kg) was orally administered 3 times during 14 days. In the PCA, AEDKC was orally treated 1 h before the antigen challenge. The control drug dexamethasone was used to compare the effectiveness of AEDKC. For *in vitro*, IgE-stimulated RBL-2H3 cells and primary cultured peritoneal mast cells were used to determine the role of AEDKC (0.01–1 mg/mL).

**Results:** Oral administration of AEDKC dose dependently suppressed rectal temperature decrease and increases in serum histamine, total IgE, OVA-specific IgE, and interleukin (IL)-4 in the ASA. In the PCA, AEDKC reduced Evans blue pigmentation. Compared to dexamethasone (10 mg/kg), AEDKC (100 mg/kg) showed similar inhibitory effects *in vivo*. AEDKC concentration dependently suppressed the release of histamine and β-hexosaminidase through the reduction of intracellular calcium in mast cells. In addition, AEDKC decreased the expression and secretion of tumour necrosis factor-α and IL-4 by the reduction of nuclear factor-κB. The inhibitory potential of AEDKC (1 mg/mL) was similar with dexamethasone (10 μM) *in vitro*.

**Conclusions:** We suggest that AEDKC may be a potential candidate for the treatment of mast cell-mediated allergic diseases.

## Introduction

*Diospyros kaki* L. (Ebenaceae), known as oriental persimmon, is widely distributed throughout several countries, including Korea, Japan, and China. The medicinal parts of *D. kaki* include the bark, root, leaves, seeds, flowers, and fruits. The traditional Chinese medicine Shitei-To, which is a mixture of extracts from three medicinal herbs, Shitei (*D. kaki* calyx), Shokyo (*Zingiberis rhizoma*), and Choji (*Caryophylli flos*), has long been used for the treatment of hiccups in Japan and China (Minami et al. 2000). *D. kaki* calyx contains various biological compounds, including gallic acid, β-sitosterol, trifolin, kaempferol, quercetin, and tannins (Sunity and Himanshu 2011). Tannins are a large group of polyphenolic compounds that are encountered in daily life owing to their wide distribution in medicinal plants and presence in food and beverages (Okuda et al. 1989). Previous studies have reported that apple-condensed tannins inhibit the activation of mast cells and subsequent histamine release (Kanda et al. 1998; Tokura et al. 2005).

Immediate-type hypersensitivity (type I allergic reaction) results in anaphylaxis induced by the degranulation of mast cells and basophils, and manifests as allergy, atopic dermatitis, and asthma (Galli et al. 2008). It is well known that immunoglobulin (Ig) E is particularly important in the type I allergic reaction. IgE molecules bind to FcɛRI, which results in the cross-linking of receptors and activation of mast cells (Galli and Tsai 2012). Mast cell activation leads to cellular calcium increase and the degranulation of mediators implicated in allergic reactions (Je, Choi, et al. 2015). Mast cells release preformed and newly synthesized mediators such as histamine, proteases, leukotrienes, and prostaglandins (Moon et al. 2014). Among these, histamine is regarded as a principal mediator of allergic reaction (Kim et al. 2017). β-hexosaminidase is also stored in the secretory granules of mast cells and is released concomitantly with histamine following immunological activation of the cells (Matsuda et al. 2016). Ovalbumin (OVA)-induced active systemic anaphylaxis (ASA) and IgE-mediated passive cutaneous anaphylaxis (PCA) are appropriate *in vivo* models for immediate-type hypersensitivity (Kim et al. 2014; Ribeiro-Filho et al. 2014). In both models, a typical allergic response is triggered by mast cell degranulation after antigen challenge.

The activation of mast cells induces the mobilization of internal calcium, which causes the release of inflammatory cytokines followed by the activation of nuclear factor (NF)-κB. NF-κB is essential for the expression of many of the genes involved in the immune and inflammatory responses (Ono et al. 2015). An increase in NF-κB activity, associated with the secretion of high levels of tumour necrosis factor (TNF)-α, has been observed in the allergic inflammatory response. TNF-α is a key mediator in many cytokine-dependent inflammatory events and is released by the allergic response from mast cells via IgE-dependent mechanisms (Thomas 2001). Interleukin (IL)-4 also plays an important role in establishing chronic allergic inflammation (Saggini et al. 2011). Therefore, the inhibition of TNF-α and IL-4 is regarded as the most important step in the treatment of allergic inflammation.

This study assesses the effects of aqueous extract of *D. kaki* calyx (AEDKC) on the immediate-type hypersensitivity. In addition, we investigated the anti-allergic effects related to the inhibition of degranulation and inflammatory cytokine expression in mast cells.

## Materials and methods

### Reagents and cell culture

Anti-dinitrophenyl (DNP) IgE, DNP-human serum albumin (HSA), ovalbumin (OVA), dexamethasone (Dexa), Histodenz, calcium ionophore A23187, and *o*-phthaldialdehyde were purchased from Sigma-Aldrich (St. Louis, MO). Alum adjuvant was procured from Thermo Scientific (Waltham, MA). RBL-2H3 and rat peritoneal mast cells (RPMCs) were grown at 37 °C in an atmosphere of 5% CO_2_ in Dulbecco’s modified Eagle’s medium and α-minimum essential medium (Gibco, Grand Island, NY), respectively, supplemented with 100 units/mL penicillin/streptomycin and heat-inactivated 10% foetal bovine serum (Gibco).

### Preparation of AEDKC

*D. kaki* calyx was purchased from the Bohwa Dang, oriental drug store (Jeonju, Korea) and identified by Professor D.K. Kim at College of Pharmacy, Woosuk University. A voucher specimen (number WSP-15-098) was deposited at the Herbarium of the College of Pharmacy, Woosuk University. The sample was extracted with purified water at 70 °C for 5 h (2 times) in water bath. Then, the extract was filtered, lyophilized, and then kept at 4 °C. The yield of dried extract from starting crude materials was about 10.1%.

### Animals

Male imprinting control region (ICR) mice (6-week-old) and Sprague Dawley (SD) rats (10-week-old) were purchased from Dae-Han Experimental Animal Center (Daejeon, Korea). All animals had *ad libitum* access to standard rodent chow and filtered water. Throughout the study, the animals were housed in a laminar air flow room in a controlled environment (temperature, 22 ± 2 °C; relative humidity, 55 ± 5%; 12-h light/dark cycle). The care and treatment of the animals were in accordance with the guidelines established by the Public Health Service Policy on the Humane Care and Use of Laboratory Animals and were approved by the Institutional Animal Care and Use Committee of Kyungpook National University (IRB #2016-0050).

### Active systemic anaphylaxis (ASA)

Thirty mice were divided into six groups (PBS only, OVA mixture only, OVA mixture and AEDKC at 1, 10, 100 mg/kg, and Dexa at 10 mg/kg). Mice (*n* = 5/group) were sensitized with the OVA mixture (100 μg of OVA and 2 mg of alum adjuvant in 200 μL of phosphate-buffered saline [PBS]) by intraperitoneal injection on day 0 and day 7. Subsequently, 1–100 mg/kg body weight (BW) AEDKC and Dexa was orally administered on days 9, 11, and 13. On day 14, 200 μg of OVA was intraperitoneally injected and rectal temperature was measured every 10 min for 90 min. After 90 min, a blood sample was obtained from the abdominal artery of each mouse.

### Passive cutaneous anaphylaxis (PCA)

An IgE-mediated PCA model was established as previously described (Je, Kim, Park, et al. 2015). Thirty mice were divided into six groups (PBS only, DNP-HSA mixture only, DNP-HSA mixture and AEDKC at 1, 10, 100 mg/kg, and Dexa at 10 mg/kg). Anti-DNP IgE (0.5 μg) was injected into the mouse ear, and 1 h prior to antigen challenge, AEDKC was administered at doses of 1, 10, and 100 mg/kg BW. After 48 h, each mouse received antigen challenge through the injection of 1 μg of DNP-HSA containing 4% Evans blue (1:1) into the tail vein. Thirty minutes after the challenge, the mice were killed, and the ears were removed for measurement of the pigment area. The amount of dye was determined after extraction with 1 mL of 1 M KOH and 9 mL of a 5:13 mixture of acetone and phosphoric acid. The absorbance intensity of the extracted solution was measured spectrophotometrically at 620 nm.

### Preparation of RPMCs

Peritoneal cells were isolated from SD rats as previously described (Kim et al. 2014). In brief, the rats were anesthetized with CO_2_ and injected with 20 mL of Tyrode’s buffer A into the peritoneal cavity, followed by gentle massage of the abdomen for approximately 90 s. The peritoneal cavity was carefully opened, and the fluid containing the peritoneal cells was aspirated using a Pasteur pipette. The cells were collected after centrifugation at 150 *g* for 10 min at room temperature and then resuspended in 1 mL of Tyrode’s buffer A. This suspension was layered on 2 mL of 0.235 g/mL Histodenz solution and centrifuged at 400 *g* for 15 min at room temperature in order to separate the mast cells from the other major components of rat peritoneal cells, i.e., macrophages and small lymphocytes. The cells at the buffer–Histodenz interface were discarded, and the cells in the pellet were washed and resuspended. Toluidine blue staining determined a purity of approximately 95% for the mast cell preparation, and trypan blue staining revealed more than 97% of the cells were viable.

### Cell viability

Cell viability was determined by colorimetric analysis using 3-(4,5-dimethylthiazol-2-yl)-2,5-diphenyltetrazolium bromide (MTT) (Je, Kim, Kim, et al. 2015). RBL-2H3 cells (6 × 10^4^ cells/well in a 96-well plate) were treated with AEDKC for 12 h, followed by incubation with MTT reagent for 2 h. The formed formazan crystals were dissolved in dimethyl sulphoxide and the absorbance was read at 570 nm using a spectrophotometer (Molecular Devices, Sunnyvale, CA).

### β-Hexosaminidase

Anti-DNP IgE (100 ng/mL)-sensitized RBL-2H3 cells (5 × 10^5^ cells/well in 12-well plates) were washed three times in PBS, treated with AEDKC for 1 h, and then stimulated with DNP-HSA (100 ng/mL) for 4 h. After incubation, the cells were separated from the media by centrifugation at 150 *g* for 5 min at 4 °C; subsequently, 40 μL of the supernatant was transferred to a 96-well plate and incubated at 37 °C for 1 h with 40 μL of 0.1 M citrate buffer (pH 4.5) containing 1 mM 4-nitrophenyl-*N*-acetyl-β-d-glucosaminide. The cells were lysed with 0.5% Triton X-100 and the supernatant was removed for the measurement of total β-hexosaminidase activity. The absorbance was measured with a spectrophotometer (Molecular Devices) at 405 nm.

### Histamine

Following centrifugation of the blood samples at 400 *g* for 15 min at 4 °C, the serum was collected. Anti-DNP IgE (50 ng/mL)-sensitized RBL-2H3 cells (5 × 10^5^ cells/well in a 12-well plate) were washed three times in PBS, treated with AEDKC for 1 h, and then stimulated with DNP-HSA (100 ng/mL) for 4 h. Isolated RPMCs were seeded into 24-well plates (2 × 10^4^ cells) in the presence of anti-DNP IgE (50 ng/mL). After 24 h, the cells were washed with PBS, treated with AEDKC for 1 h, and then stimulated with DNP-HSA (100 ng/mL) for 30 min. The cells were separated from the medium by centrifugation at 150 *g* for 5 min at 4 °C. To measure histamine in the serum and separated medium, 0.1 N HCl and 60% perchloric acid were added to the samples, which were then centrifuged. The supernatant was transferred to a 1.5-mL Eppendorf tube; 5 M NaCl, 5 N NaOH, and *n*-butanol were added; and the solution was vortexed and centrifuged again. The supernatant was mixed with 0.1 N HCl and *n*-heptane and then centrifuged. Histamine in the aqueous layer was measured using the *o*-phthaldialdehyde spectrofluorometric procedure as previously described (Je, Kim, Park, et al. 2015). Fluorescence intensity was detected using a fluorescence plate reader (Molecular Devices) at an excitation wavelength of 380 nm and an emission wavelength of 440 nm.

### Intracellular calcium

Intracellular calcium was measured using the fluorescent indicator Fluo-3/AM (Invitrogen, Carlsbad, CA) (Kim et al. 2014). RBL-2H3 cells (6 × 10^4^ cells/well in 96-well plates) were sensitized by overnight exposure to anti-DNP IgE (50 ng/mL). The cells were then incubated with Fluo-3/AM for 1 h at 37 °C and washed with Tyrode’s buffer to remove the dye from the cell surface. The cells were then pretreated with or without AEDKC for 1 h prior to antigen challenge with DNP-HSA (100 ng/mL). BAPTA-AM (Calbiochem, La Jolla, CA), a calcium chelator, was used as the positive control. Fluorescent intensity was detected using a fluorescent plate reader at an excitation wavelength of 485 nm and an emission wavelength of 510 nm. The intracellular calcium level in untreated control cells was assigned a relative absorbance value of 1.

### Enzyme-linked immunosorbent assay (ELISA)

Cytokine and IgE levels were measured by ELISA (Kim et al. 2014). To isolate the serum, the mouse blood was clotted at room temperature and centrifuged at 400 *g* for 15 min at 4 °C. RBL-2H3 cells (5 × 10^5^ cells/well in a 12-well plate) were sensitized with overnight exposure to anti-DNP IgE (50 ng/mL). The cells were pretreated with AEDKC for 1 h prior to a challenge with DNP-HSA (100 ng/mL) for 6 h. The ELISA was performed on a 96-well Nunc Immuno Plate using specific kits (BD Biosciences, San Diego, CA) according to the manufacturer’s protocol. Before the detection of OVA-specific IgE, the plate was coated with OVA instead of a capture antibody. After the substrate reaction was terminated, the absorbance was measured using a spectrophotometer (Molecular Devices) at a wavelength of 450 nm.

### qPCR

After stimulation with DNA-HSA with or without AEDKC, cells were seeded at a density of 5 × 10^5^ cells/well in a 12-well plate. Total cellular RNA was isolated using a RNAiso Plus kit (Takara Bio, Shiga, Japan) according to the manufacturer’s protocol. The first strand complementary DNA (cDNA) was synthesized using a PCR kit (Thermo Scientific). The primer sets were chosen by the Primer3 program (Whitehead Institute, Cambridge, MA). The cycle number was optimized to ensure product accumulation was in the exponential range. qPCR was carried out using a Thermal Cycler Dice TP850 (Takara Bio) according to the manufacturer’s protocol. Briefly, 2 μL of cDNA (100 ng), 1 μL of sense and antisense primer solution (0.4 μM), 12.5 μL of SYBR Premix Ex Taq (Takara Bio), and 9.5 μL of nuclease free water were mixed together to obtain a final volume of 25 μL in each reaction tube. Relative quantification of mRNA expression was performed using the TP850 software. The conditions used for PCR were as previously described (Bae et al. 2011).

### Western blotting

Nuclear and cytosolic proteins were extracted as previously described (Choi et al. 2013). Anti-DNP IgE (50 ng/mL)-sensitized RBL-2H3 cells (1.5 × 10^6^ cells/well in a 6-well plate) were washed three times with PBS, treated with AEDKC for 1 h, and then stimulated with DNP-HSA (100 ng/mL) for 1 h. Equal amounts of cellular protein were electrophoresed using a 8% sodium dodecyl sulphate–polyacrylamide gel and transferred to a nitrocellulose membrane. After blocking, the membrane was incubated with a primary antibody against the target and then with anti-IgG horseradish peroxidase-conjugated secondary antibody. The following antibodies were purchased from Santa Cruz Biotechnology: NF-κB (sc-109, rabbit polyclonal, 1:1000), IκBα (sc-371, rabbit polyclonal, 1:1000), β-actin (sc-8432, mouse monoclonal, 1:1000), and lamin B (sc-6217, goat polyclonal, 1:1000). Immunodetection was carried out using a chemiluminescent substrate (Thermo Scientific).

### Statistics

Statistical analyses were performed using SAS statistical software (SAS Institute, Cary, NC). Treatment effects were analyzed using ANOVA followed by Duncan’s multiple range tests. A value of *p* < 0.05 was considered statistically significant.

## Results

### Effects of AEDKC on systemic and local anaphylaxis

**Figure 1.**
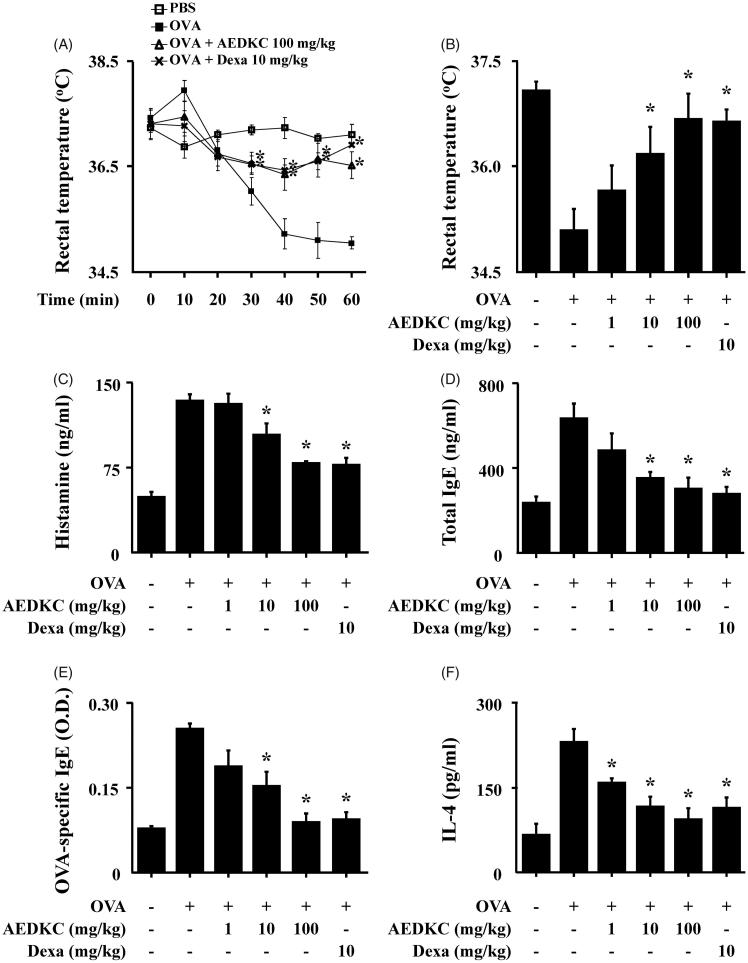
Effects of AEDKC on ovalbumin-induced active systemic anaphylaxis. The induction of systemic anaphylaxis and oral administration of drugs, including AEDKC and Dexa, are described in the Materials and methods section. Blood was obtained from the abdominal artery of each mouse and measurements of serum histamine, total IgE, OVA-specific IgE, and IL-4 were taken. (A) Rectal temperature was measured every 10 min for 1 h. (B) Rectal temperature of the mice at 50 min. (C–F) Serum levels of histamine, total IgE, OVA-specific IgE, and IL-4. Graph data represent the mean ± SD (*n* = 5/group) of two independent experiments. *p < 0.05 compared with the OVA-challenged group. Dexa: dexamethasone.

The systemic anaphylaxis model is widely used to examine immediate-type hypersensitivity, which is strongly associated with mast cells (Marone et al. 2002). Mice were sensitized through repetitive administrations of OVA with alum adjuvant, and anaphylaxis was induced by challenge with OVA. After an intraperitoneal injection of OVA, mice were monitored for 90 min. Over a period of 20–60 min, mouse body temperature was decreased, while the serum histamine level was considerably increased. The observed decrease in rectal temperature was reduced by oral administration of AEDKC, and the serum histamine level was also diminished (Figure 1(A–C)). Total serum IgE, OVA-specific IgE, and IL-4 levels were increased after the challenge with OVA and decreased by AEDKC (Figure 1(D–F)).

**Figure 2.**
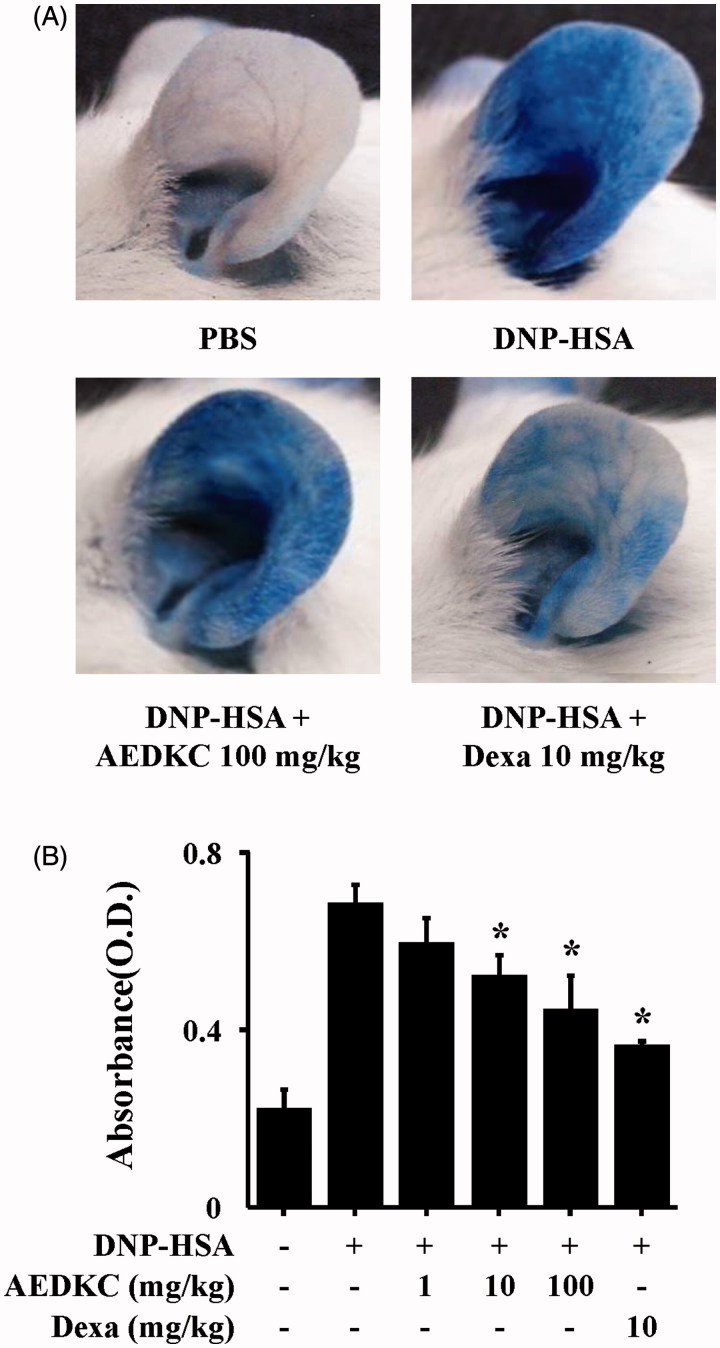
Effects of AEDKC on IgE-mediated passive cutaneous anaphylaxis. Mouse ear skin (*n* = 5/group) was sensitized with an intradermal injection of anti-DNP IgE (0.5 μg/site) for 48 h. AEDKC and Dexa were orally administered 2 h before the intravenous injection of DNP-HSA (1 mg/mouse) and 4% Evans blue (1:1) mixture. Thirty minutes later, the ears were collected to measure pigmentation. The dye was extracted as described in the Materials and methods section and detected using a spectrophotometer. (A) Representative photographic images of ears. (B) Graph data represent the mean ± SD (*n* = 5/group) of two independent experiments. *p < 0.05 compared with the DNP-HSA-challenged group. Dexa: dexamethasone.

To examine the effects of AEDKC on IgE-mediated allergic reaction *in vivo*, PCA model was used. The PCA model is one of the most appropriate *in vivo* models of local allergic reaction. After injection of 4% Evans blue dye mixed with antigen, the PCA reaction site showed a marked increase in vascular permeability, as indicated by the extravasation of Evans blue dye. Oral administration of AEDKC (1–100 mg/kg BW) visibly reduced PCA reaction (Figure 2).

### Effects of AEDKC on intracellular calcium level and degranulation in mast cells

**Figure 3.**
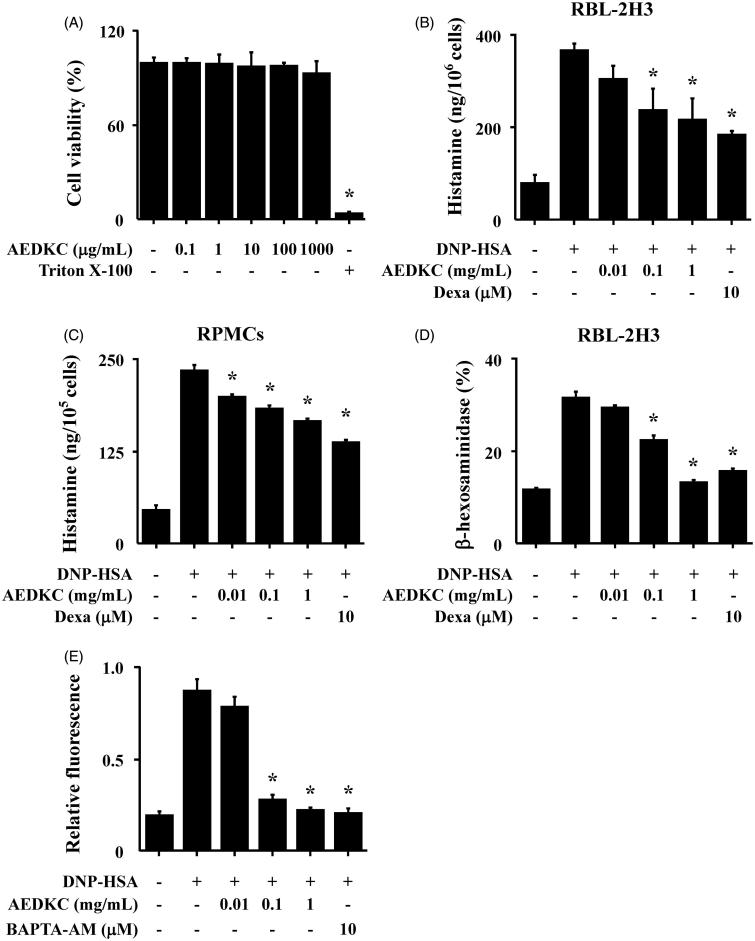
Effects of AEDKC on intracellular calcium and mast cell degranulation. (A) RBL-2H3 cells (6 × 10^4^/well) were pretreated with or without AEDKC for 12 h and then incubated with 1 mg/mL MTT for 2 h. The absorbance intensity was measured using a spectrophotometer. (B,C) RBL-2H3 cells (5 × 10^5^/well) and RPMCs (2 × 10^4^/well) were sensitized with anti-DNP IgE (50 ng/mL). After overnight incubation, the cells were pretreated with or without drugs, including AEDKC and Dexa, for 1 h, and then challenged with DNP-HSA (100 ng/mL). (D) The level of β-hexosaminidase was measured using β-hexosaminidase substrate buffer. RBL-2H3 cells (6 × 10^4^/well) were sensitized with anti-DNP IgE. (E) After overnight anti-DNP IgE incubation, cells were incubated with Fluo-3/AM for 1 h, treated with or without AEDKC for 1 h, and then challenged with DNP-HSA. Intracellular calcium was detected using a fluorescent plate reader. BAPTA-AM, a calcium chelator, was used as the positive control. Graph data represent the mean ± SD of three independent experiments. *p < 0.05 compared with the DNP-HSA-challenged group. Dexa: dexamethasone.

First, the cytotoxicity of AEDKC was tested by MTT assay. RBL-2H3 cells were treated with various concentrations (0.1–1000 μg/mL) of AEDKC and incubated for 12 h. Below 1000 μg/mL of AEDKC, cell viability was unaltered (Figure 3(A)). We assessed the effect of AEDKC on the degranulation of mast cells by the measurement of histamine and β-hexosaminidase release. DNP-HSA-challenged RBL-2H3 cells released high levels of histamine and β-hexosaminidase, and this release was dose dependently decreased by AEDKC treatment (Figure 3(B,D)). The inhibitory effect of AEDKC was confirmed in primary cultured RPMCs (Figure 3(C)). To investigate the mechanism by which AEDKC causes the reduction of histamine release, we assayed the intracellular calcium. The inhibition of calcium influx by AEDKC was examined using the fluorescent indicator Fluo-3/AM. The incubation of RBL-2H3 cells with AEDKC (0.1–1 mg/mL) dose dependently decreased the intracellular calcium level (Figure 3(E)).

### Effects of AEDKC on NF-κB activation and inflammatory cytokine expression in mast cells

**Figure 4.**
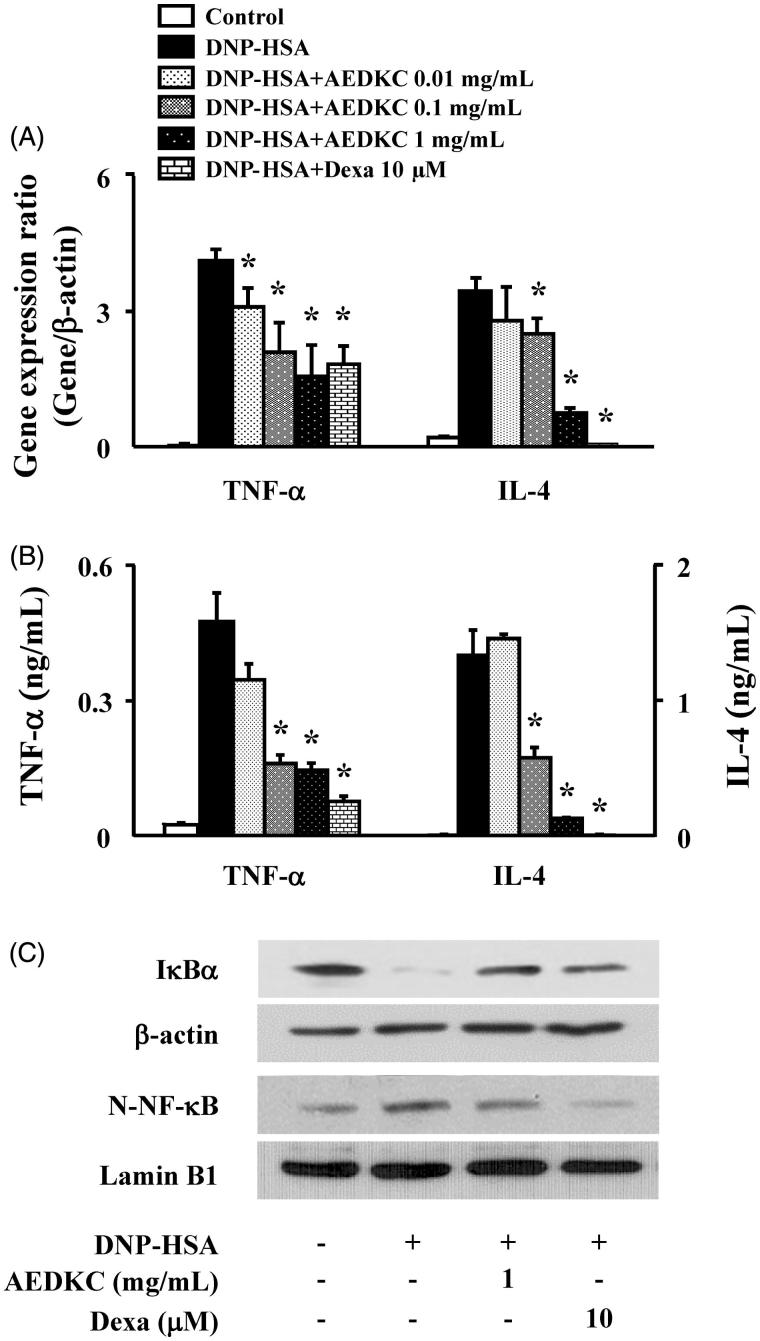
Effects of AEDKC on inflammatory cytokines and NF-κB activation in mast cells. RBL-2H3 cells (5 × 10^5^/well) were sensitized with anti-DNP IgE (50 ng/mL). After overnight incubation, the cells were pretreated with or without drugs, including AEDKC and Dexa, for 1 h, and then challenged with DNP-HSA (100 ng/mL). (A) The gene expression of inflammatory cytokines was determined by qPCR. (B) The secretion of inflammatory cytokines was measured by ELISA. Graph data represent the mean ± SD of three independent experiments. (C) NF-κB activation was assayed by Western blot (N: nuclear). β-actin and lamin B were used as loading controls. The bands are representative of three independent experiments. *p < 0.05 compared with the DNP-HSA-challenged group. Dexa: dexamethasone.

We tested the effect of AEDKC on the expression of pro-inflammatory cytokines, such as TNF-α and IL-4, in RBL-2H3 cells. RBL-2H3 cells stimulated with DNP-HSA for 1 h expressed all cytokines at high levels. AEDKC (0.1–1 mg/mL) concentration dependently inhibited the gene expression and secretion of cytokines (Figure 4(A,B)). To determine the suppression mechanism of the inflammatory cytokines, the activation of NF-κB was evaluated. In our results, degradation of IκBα and nuclear translocation of NF-κB were observed in the RBL-2H3 cells stimulated with DNP-HSA; however, these effects were significantly suppressed by AEDKC (Figure 4(C)).

## Discussion

Immediate-type hypersensitivity is a life-threating syndrome triggered by the sudden systemic release of inflammatory mediators such as cytokines, ILs, leukotrienes, and prostaglandins, which are secreted from immune cells, i.e., eosinophils, basophils, and mast cells. In particular, mast cells are important in the control of allergic reactions (Amin 2012). A recent study revealed that mast cell-released histamines, cytokines, and proteases perform crucial roles during the allergic inflammation in mast cell-deficient mice (Lee 2016). Therefore, the development of drug candidates that inhibit mast cell activation is important for human health.

Phytochemicals have traditionally been used as a remedy for various diseases in many Asian countries. *D. kaki* is used for the treatment of asthma, chronic bronchitis, allergies, and coughing (Tetsumura et al. 2015). Its leaves have been shown to exert haemostatic activity, blood pressure reduction, and antimicrobial and neuroprotective effects (Jung et al. 2015). Especially, coussaric acid and betulinic acid, both triterpenoid compounds, were obtained from *D. kaki* leaf extracts through bioassay-guided isolation and showed anti-inflammatory effects via inhibition of the NF-κB pathway, providing important information on their anti-inflammatory mechanism (Kim et al. 2016). In our previous study, *D. kaki* has shown anti-allergic inflammatory effect (Kim et al. 2013). In addition, quercetin isolated from *D. kaki* calyx has been shown to possess anti-inflammatory potential (Cho et al. 2016). However, the anti-allergic effects of *D. kaki* calyx have not been elucidated.

Experiments in the ASA model demonstrated that the anaphylactic degranulation of mast cells was inhibited by AEDKC treatment. The AEDKC-mediated reduction in pigmentation observed in the PCA model was also considered to arise from the suppression of mast cell degranulation. Indeed, this was supported by the observation that AEDKC significantly inhibited degranulation of the mast cell line (RBL-2H3) and primary cultured mast cells (RPMCs). Furthermore, AEDKC resulted in significant inhibition of the gene expression of TNF-α and IL-4, which was induced by antigen challenge in mast cells. It is now well established that mast cells are the source of several multifunctional cytokines (Shuang Wang 2016). Considerable evidence indicates that IL-4 from mast cells may be implicated in the pathogenesis of allergic inflammations such as allergy, asthma, and rhinitis. IL-4, a pleiotropic cytokine, is known to be required for B cell isotype switching in favour of IgE production (Moens and Tangye 2014; Jeon et al. 2015). Therefore, it has been suggested that IL-4 is essential for establishing chronic allergic inflammation. This was supported by evidence that ear swelling in the late phase reaction was significantly suppressed by treatment with anti-IL-4 monoclonal antibody in the ASA model. TNF-α is a key mediator in many cytokine-dependent inflammatory events. TNF-α is released by the allergic response of both mast cells and macrophages through IgE-dependent mechanisms. It has been established that TNF-α induces chemotaxis of neutrophils and T cells, enhances cytotoxicity of macrophages, and stimulates the expression of adhesion molecules from endothelial cells. A previous study also indicated that TNF-α released from mast cells partially inhibited apoptosis, which was responsible for eosinophil survival, and contributed to chronic inflammation (Shuang Wang 2016). Consequently, our results suggest that AEDKC affects the acute allergic reaction, as well as chronic inflammation, through the regulation of cytokine expression in mast cells.

The degranulation of mast cells is regulated by the intracellular calcium (Vig et al. 2008). Depletion of intracellular calcium blocked the expression of IgE-induced TNF-α in mast cells (Manikandan et al. 2012; Je et al. 2013). In our experiments, AEDKC decreased the level of intracellular calcium in mast cells. We suggested that the AEDKC-induced inhibition of inflammatory cytokines resulted from the reduction of intracellular calcium. In addition, intracellular calcium is important in the translocation of NF-κB (Lian et al. 2015), which is a transcription factor for a variety of genes. Many of these genes encode molecules that are important in inflammatory processes, such as cytokines and adhesion molecules. The role of NF-κB in the regulation of cytokine production in allergic inflammation has been characterized (Bae et al. 2011). In our study, AEDKC decreased the nuclear translocation of NF-κB and NF-κB-dependent gene expression. These data demonstrate that AEDKC attenuated allergic inflammation through the inhibition of NF-κB.

Collectively, we provided evidence for the contribution of AEDKC in the prevention or treatment of mast cell-mediated immediate-type hypersensitivity. Our results demonstrated the mechanisms responsible for the anti-allergic activity of AEDKC. Therefore, we propose that AEDKC may be a candidate to treat the allergic response from the activation of mast cells.
